# Prognostic role of hormone receptors in endometrial cancer: a systematic review and meta-analysis

**DOI:** 10.1186/s12957-015-0619-1

**Published:** 2015-06-25

**Authors:** Yanli Zhang, Dong Zhao, Changguo Gong, Fengmei Zhang, Jing He, Wei Zhang, Yulan Zhao, Jing Sun

**Affiliations:** Department of Minimally Invasive Gynecologic Surgery, Shanghai First Maternity and Infant Hospital, Tongji University School of Medicine, Changle Road #536, Shanghai, 200040 People’s Republic of China; School of Life Science, East China Normal University, North Zhongshan Road #3663, Shanghai, People’s Republic of China; Institutes for Advanced Interdisciplinary Research, East China Normal University, Shanghai, People’s Republic of China

**Keywords:** Endometrial cancer, Estrogen receptor, Progesterone receptor, Human epidermal growth factor receptor 2, Prognosis

## Abstract

**Background:**

The aim of this study was to summarize the global predicting role of hormone receptors for survival in endometrial cancer.

**Methods:**

Eligible studies were identified and assessed for quality through multiple search strategies. Data were collected from studies comparing overall survival (OS), cancer-specific survival (CSS), or progression-free survival (PFS) in patients with elevated levels of estrogen receptor (ER), progesterone receptor (PR), or human epidermal growth factor receptor 2 (HER2) with those in patients with lower levels. The combined hazard ratios of ER, PR, and HER2 for survival were calculated.

**Results:**

A total of 98 studies were included for meta-analysis (44 for ER, 38 for PR, and 16 for HER2). Higher levels of either ER or PR could significantly indicate better survival. The pooled hazard ratios (HRs) of ER for OS, CSS, and PFS were 0.75 (95 % CI, 0.68–0.83), 0.45 (95 % CI, 0.33–0.62), and 0.66 (95 % CI, 0.52–0.85), respectively. The combined HRs of PR for OS, CSS, and PFS reached 0.63 (95 % CI, 0.56–0.71), 0.62 (95 % CI, 0.42–0.93), and 0.45 (95 % CI, 0.30–0.68), respectively. In contrast, elevated levels of HER2 could predict worse outcome with a HR of 1.98 (95 % CI, 1.49–2.62) for OS, and a HR of 2.26 (95 % CI, 1.57–3.25) for PFS.

**Conclusions:**

In patients with endometrial cancer, higher level of ER and PR predicted favorable survival, and increased level of HER2 was associated with poorer survival. All of the three hormone receptors had prognostic value for survival.

## Background

Endometrial cancer (EC) is the fourth most common malignancy in women and the most common gynecologic cancer [1], and in 2014, 52,630 new cases was diagnosed with an estimated 8590 deaths predicted in the USA alone [2]. The incidence of EC is also increasing in developing countries in the past decades [3, 4]. Overall, the 5-year survival rates for EC are approximately 78–90 % for stage I, 74 % for stage II, 36–57 % for stage III, and 20 % for stage IV [5]. Additionally, women with metastatic disease have only a median survival of 7–12 months [6]. Such poor outcomes raise an urgent requirement that more accurate prognosis and predictive markers should be applied for EC to guide the therapy and monitor the disease progress for individual patients.

Endometrial cancer is the most common genital tract malignancy in women and consists of two major histological types, endometrioid endometrial cancer, and non-endometrioid endometrial cancer including high-risk malignancies such as serous papillary and clear cell carcinoma. Endometrioid endometrial carcinoma is the most common form, accountable for more than 75–90 % of all cases of endometrial cancer [7].

Besides conventional clinical or pathological features, some biological molecules have been proposed as prognostic biomarkers in EC, such as P53, KRAS, PTEN, EGFR, FGFR, estrogen receptors (ER), progesterone receptors (PR), human epidermal growth factor receptor 2 (HER2), and so on [8]. Among them, hormone receptors are attractive because of their physiological functions. Through binding to their receptors, estrogen drives epithelial proliferation, and progesterone inhibits growth and causes cell differentiation. Interestingly, women who ovulate and produce progesterone almost never get endometrial cancer. Oppositely, disruption of the functions of hormone receptors can lead to several types of malignancies [9]. Due to higher response rates reported for hormone receptor-positive tumors, these receptors are currently considered to be important therapeutic targets and markers for the choice of treatment [10]. HER2 is a member of the human epidermal growth factor receptor tyrosine kinase family, which regulates many processes that can promote tumor cell proliferation and survival [11]. HER2 pathway, which may interact with ER, is one of the most important pathways that have been implicated in the development of endocrine resistance in breast cancer. With the development of molecular biology and immunologic method, all of the three hormone receptors have been introduced to refine outcome prediction of female cancers, such as breast cancer, ovarian cancer, and endometrial cancer.

Our previous meta-analysis reported that higher level of PR predicted favorable survival, and elevated level of HER2 was associated with worse survival in ovarian cancer. Furthermore, ER-β may be a potentially strong predictor for better outcome [12]. A comparable situation may also exist in research of EC, another malignant tumor affected by the interaction between steroid hormones and their respective receptors. Although a pile of clinical studies on prognostic value of ER, PR, and HER2 expression levels in EC has also been done, no clear conclusion could be drawn to date. In 1985, Creasman et al. reported that hormone receptor expression correlates with disease-free survival in stages I and II endometrial carcinoma [13]. However, inconsistent results were obtained in the followed studies [14–17]. For example, some studies showed that elevated levels of ER or PR could significantly predict favorable outcome [18, 19], whereas some other studies showed insignificant results [20–22]. Moreover, some studies suggested that elevated HER2 level was associated with poorer survival, whereas other studies could not draw such significant conclusion [20, 22].

Therefore, it is timely and necessary to analyze globally the prognostic value of hormone receptors in a larger population. In this study, we seek to conduct a meta-analysis to evaluate the overall risk of hormone receptors for endometrial cancer survival. We discussed endometrial carcinoma and uterine papillary serous carcinoma in this text.

## Methods

We performed meta-analysis following the guidelines of the Meta-analysis of Observational Studies in Epidemiology group (MOOSE) [23].

### Search strategy

We carefully searched online PubMed and EMBASE from 1979 to May 2014 to identify relevant studies. Three distinct sets of key words were used simultaneously in each set, namely, “estrogen receptor and endometrial cancer prognosis,” “progesterone receptor and endometrial cancer prognosis,” and “human epidermal growth factor receptor 2 and endometrial cancer prognosis.” Studies were considered eligible if they met the following criteria: (1) they measured preoperative ER, PR, or HER2 values; (2) they evaluated the potential association between preoperative ER, PR, or HER2 levels and the outcome of endometrial cancer; (3) their study was retrospective or prospective in design; and (4) the median period of follow-up was no shorter than 6 months. Articles were excluded based on the following criteria: (1) review articles or letters, (2) non-English articles, (3) laboratory studies, and (4) absence of key information such as sample size, hazards ratio (HR), 95 %confidence interval (CI), and *P* value.

Titles, abstracts, full texts, and reference lists of all of the identified reports were examined independently by three reviewers (Zhang Y, Gong C, and Zhang F). These extracted data have been double-checked by each other. Disagreements were resolved by consensus between the three readers or consultation with a fourth reviewer (Zhao Y or Zhao D). In addition, a manual search was conducted using references from the relevant literature, including all of the identified studies, reviews, and editorials. We e-mailed to the authors of studies for additional information and the data needed for the meta-analytic calculations. When duplicate studies were retrieved, we included in our systematic review the study having reported HRs or involving more patients (usually the latest). This was performed to avoid overlapping between cohorts and overestimation of the overall HR.

### Quality assessment

**Fig. 1:**
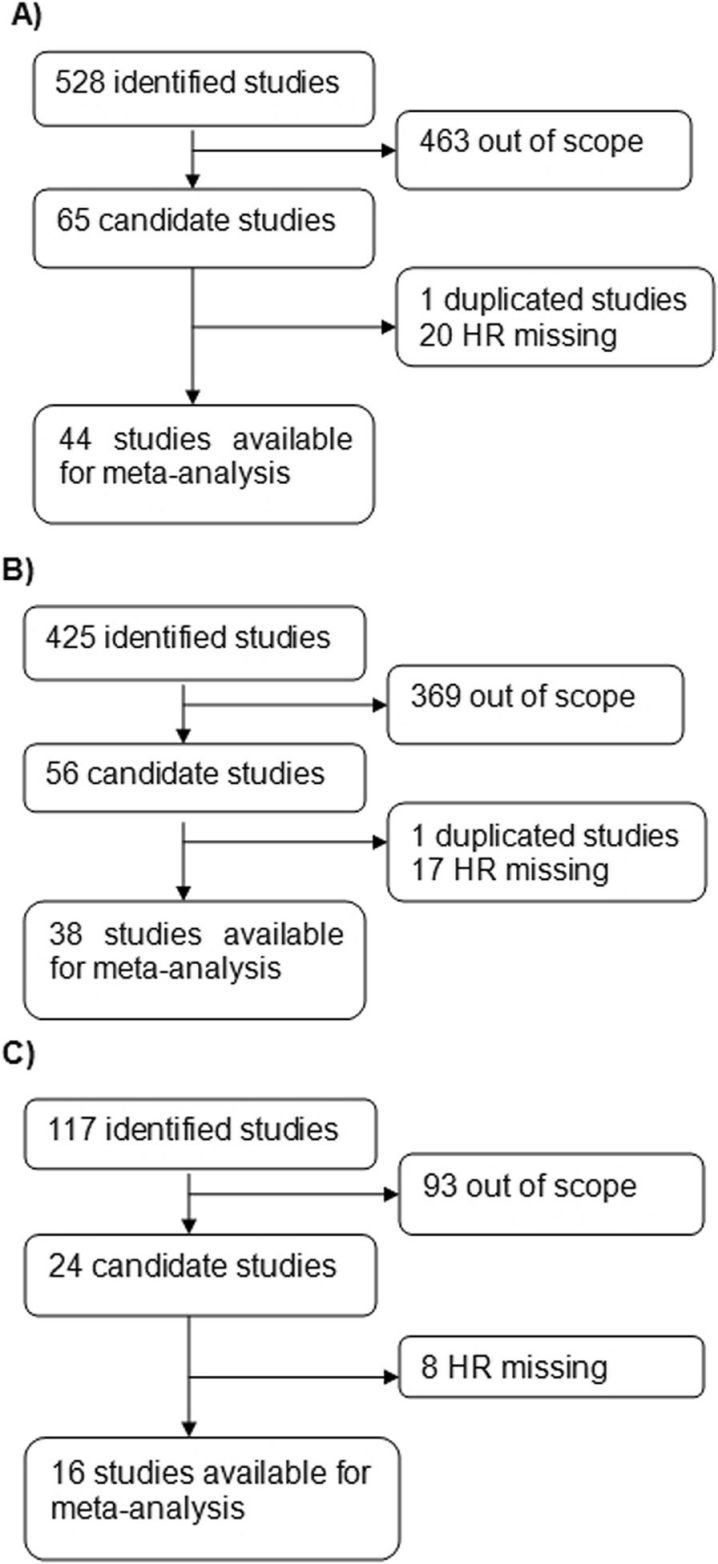
Flow diagram of the study selection process. **a** ER studies. **b** PR studies. **c** HER2 studies

According to a critical review checklist of the Dutch Cochrane Centre proposed by MOOSE, we systematically assessed the quality of all the studies included [23]. The key points of the current checklist include (1) clear definition of study population; (2) clear definition of study design; (3) clear definition of outcome assessment, such as overall survival (OS), cancer-specific survival (CSS), disease-specific survival (DSS), progression-free survival (PFS), disease-free survival (DFS), or recurrence-free survival (RFS); and (4) sufficient period of follow-up. If a study does not mention all four points, it was excluded so as not to compromise the quality of the meta-analysis. A flow diagram of the study selection process is presented in Fig. 1.

### Data extraction and conversion

The extracted data elements of this review included (1) publication details: first author’s last name, publication year, and origin of the studied population; (2) study design; (3) characteristics of the studied population: sample size, age, stage of disease, or histological type; and (4) HR of elevated ER, PR, and HER2 for OS, CSS (including DSS), and PFS (including DFS and RFS), as well as their 95 % CI and *P* value. The simplest method consisted in the direct collection of HR, odds ratio or risk ratio, and their 95 % CI from the original article, with an HR of less than 1 being associated with a better outcome. If not available, the total numbers of observed deaths/cancer recurrences and the numbers of patients in each group were extracted to calculate HR .When data were only available as Kaplan-Meier curves, data were extracted from the graphical survival plots, and estimation of the HR was then performed using the described method.

### Statistical analysis

A test of heterogeneity of combined HRs was conducted using Cochran *Q* test and Higgins I-squared statistic. A *P* value of less than 0.05 was considered significant. A random-effect model (Der Simonian and Laird method) was used if heterogeneity was observed (*P* < 0.05), whereas the fixed-effect model was applied in the absence of between-study heterogeneity (*P* < 0.05). Publication bias was evaluated using the funnel plot with the Egger bias indicator test [24]. All analyses were conducted using Stata: Data Analysis and Statistical Software V10.1 (http://www.stata.com/).

## Results and discussion

A total of 528 records for ER were identified from a primary literature search in PubMed and EMBASE. After manually screening the titles, abstracts, and key data, 463 studies were excluded because they were review articles, letters, non-English articles, laboratory studies, studies with important data missing, or studies irrelevant to the current analysis. Of the 65 reports selected for detailed evaluation, 1 study was excluded for being duplicated; 20 others were excluded for lack of key data, such as HR. The final meta-analysis was carried out for the remaining 44 studies (*n* = 7119) for ER [13, 14, 16–18, 20–22, 25–61] (Fig. 1a). A similar identification process was carried out in 425 studies for PR and 117 studies for HER2. Finally, 38 studies recruiting 5502 patients for PR [13, 14, 16–18, 20–22, 25, 26, 29, 31, 32, 34–43, 50–59, 61–65] (Fig. 1b) and 16 studies recruiting 1764 patients for HER2 were included [20, 22, 33, 52, 54, 66–76] (Fig. 1c). The main features of eligible studies are summarized in Table 1. We collected data from Australia, China, England, Finland, Germany, Greece, Italy, Japan, Korea, Netherlands, Norway, Spain, Sweden, Turkey, and the USA.

**Table 1:** Summary table of the meta-analysis

A) ER								
	Country	Study design	Disease	*N*	Age (range)	Survival analysis	Hazard ratios	Follow-up, months
Athanassiadou 1999 [39]	Greece	R	EC	80	62.7 (48–82)	OS	SC	140
Backe 1997 [54]	German	R	EC	124	68 (30–94)	OS	Reported	57.6 (0.24–180)
Borazjani 1989 [40]	USA	R	EC	44	66 (36–86)	OS	SC	120
Chambers 1988-1 [51]	USA	R	EC	168	–	OS	DE	24 (1–118.8)
Covens 2011 [45]	USA	P	EC	67	–	OS, PFS	SC	36
Creasman 1985 [13]	USA	R	EC	168	63 (30–92)	DFS	DE	25 (1–74)
Engelsen 2008 [34]	Norway	R	EC	230	–	OS	SC	192
Felix 2012 [28]	USA	R	EC	199	–	OS, RFS	SC	42 (0.8–144)
Fukuda 1998 [14]	Japan	R	EEC	92	60.3 (31–86)	DFS, OS	SC, reported	61.2 (0–174)
Gates 2006 [52]	USA	R	EC	108	64.2 (27–95)	OS	DE	60
Gonzalez-Rodilla 2013 [20]	Spain	R	EC	126	65.9 (43–88)	OS	Report	70
Gul 2010 [31]	Turkey	R	EC	49	58.3 (30–81)	OS	DE	24
Huvila 2013 [61]	Finland	R	EEC	182	67 (35–93)	DFS	Reported	62.8 (4.2–84.4)
Ito 2005 [57]	Japan	R	EEC	103	57	OS	Reported	60 (2–148)
Jongen 2009 [17]	Netherlands	R	EEC	315	64.7 (32–89)	DSS, RFS, OS	SC	59.6 (0–258)
Kadar 1993 [16]	USA	R	EC	137	–	OS	DE	60
Kamat 2009 [32]	USA	R	EEC	139	63 (27–91)	DSS	Reported	24.9
Kalogiannidis 2008 [35]	Greece	R	EC	77	62.5 (35–80)	OS, CSS, DFS	DE	60 (9–120)
Kauppila 1986 [42]	Finland	R	EC	153	–	DFS, OS	SC	42 (12–96)
Krakstad 2012-primary [27]	Norway	P	EC	182	–	DSS	SC	60
Krakstad 2012-prospective [27]	Norway	P	EC	474	–	DSS	SC	60
Lenhard 2013 [59]	German	P	EC	292	65.1 (35.6–88)	OS	Reported	13.8 (13.1–14.5)
Liao 1986 [43]	USA	R	EC	75	–	OS	SC	50
Lindahl 1992 [50]	Sweden	R	EC	298	63 (36–87)	OS	DE	60
Martin 1983 [44]	Australia	P	EC	87	(48–85)	OS	SC	(8–68)
Merritt 2010 [55]	USA	R	EEC	85	63.4 (39–91)	DSS	Reported	72
Mhawech-Fauceglia 2013 [48]	USA	R	EC	316	–	OS	DE	60
Mylonas 2010 [30]	Germany	R	EEC	214	65.1 (35–88)	PFS, CSS, OS	SC	96.3 (0.03–176.8)
Palmer 1988 [41]	Australia	R	EC	351	64.5 (31–89)	OS	SC	100
Pertschuk 1996 [47]	Caucasian, Hispanic, Oriental	R	EC	78	65.5 (38–89)	OS	SC	37.5 (13–161)
Pradhan 2012 [26]	Norwegian	P	UPSC	52	72 (56–89)	OS, PFS	DE	60
Saito 2006 [56]	Japan	R	EEC	103	57	DFS, OS	Reported	60 (2–148)
Rahman 2013 [18]	Japan	R	EEC	111	60 (26–85)	PFS, OS	Reported	52 (5–139)
Salvesen 1998 [58]	Norway	P	EC	97	65 (37–92)	OS	DE	108 (60–180)
Shabani 2007 [36]	Germany	R	EC	293	64.8 (35.5–88)	PFS, CSS, OS	SC	89.6 (3.2–135.5)
Sho 2014 [60]	Japan	R	UPSC	33	69.6 (55–82)	CSS	Reported	29 (2–174)
Singh 2007 [37]	USA	P	EC	48	–	OS	Reported	19
Sivridis 2001 [38]	Greece	R	EC	164	–	OS	SC	55 (19–167)
Song 2012 [29]	Korea	R	EC	137	53.7 (30–82)	OS	Reported	60
Sun 2013 [46]	China	P	EC	73	58 (30–78)	DFS	SC	43.4 (16–91)
Voss 2011 [22]	England	P	EC	156	68.2 (37–89)	DSS, RFS	Reported	48.1 (0.1–141.5)
Wik 2013-R [49]	Norway	R	EC	266	–	DSS	SC	300
Wik 2013-P [49]	Norway	P	EC	153	–	DSS	SC	300
Zannoni 2013 [25]	Italy	P	EEA	121	59 (35–88)	DFS, OS	Reported	38 (14–91)
Zhang 2013 [53]	China	R	EC	239	54 (26–82)	DFS, OS	DE	67 (12–183)
Zou 2012 [21]	China	R	EEC	60	51.3 (30–72)	OS	Reported	45.5 (3–69.5)
B) PR								
	Country	Study design	Disease	*N*	Age (range)	Survival analysis	Hazard ratios	Follow-up, months
Athanassiadou 1999 [39]	Greece	R	EC	80	62.7 (48–82)	OS	SC	140
Backe 1997 [54]	German	R	EC	197	68 (30–94)	OS	Reported	57.6 (0.2–180)
Borazjani 1989 [40]	USA	R	EC	44	66 (36–86)	OS	SC	120
Chambers 1988-1 [51]	USA	R	EC	168	67 (49–90)	OS	DE	24 (1–118.8)
Creasman 1985 [13]	USA	R	EC	105	63 (30–92)	DFS	DE	25 (1–74)
Ehrlich 1988 [65]	USA	R	EC	174	56 (25–89)	OS	SC	27 (1–152)
Engelsen 2008 [34]	Norway	R	EC	230	–	OS	Reported	192
Fukuda 1998 [14]	Japan	R	EEC	92	60.3 (31–86)	DFS	SC	61.2 (0–174)
Gates 2006 [52]	USA	R	EC	108	64.2 (27–95)	OS	Report	60
Gonzalez-Rodilla 2013 [20]	Spain	R	EC	126	65.9 (43–88)	OS	Reported	70
Gul 2010 [31]	Turkey	R	EC	49	58.3 (30–81)	OS	DE	24
Huvila 2013 [61]	Finland	R	EEC	182	67 (35–93)	DFS	Reported	62.8 (4.2–84.4)
Ito 2005 [57]	Japan	R	EEC	103	57	DFS, OS	Reported	60 (2–148)
Jongen 2009 [17]	Netherlands	R	EEC	300	64.7 (32.0–89.0)	DSS, RFS, OS	SC, reported	59.6 (0–258)
Kadar 1993 [16]	USA	R	EC	137	–	OS	DE	60
Kalogiannidis 2008 [35]	Greece	R	EC	77	62.5 (35–80)	OS, CSS, DFS	DE	60 (9–120)
Kamat 2009 [32]	USA	R	EEC	139	63 (27–91)	DSS	report	24.9
Kauppila 1986 [42]	Finland	R	EC	153	–	DFS, OS	SC	42 (12–96)
Liao 1986 [43]	USA	R	EC	86		OS	SC	50
Lenhard 2013 [59]	German	P	EC	292	65.1 (35.6–88.1)	OS	Reported	13.8 (13.1–14.5)
Lindahl 1992 [50]	Sweden	R	EC	272	63 (36–87)	OS	DE	60
Merritt 2010 [55]	USA	R	EEC	85	63.4 (39–91)	DSS	Reported	72
Palmer 1988 [41]	Australia	R	EC	351	64.5 (31–89)	OS	SC	100
Pradhan 2012 [26]	Norwegian	P	SAC	50	72 (56–89)	OS, PFS	DE	60
Rahman 2013 [18]	Japanese	R	EEC	110	60 (26–85)	PFS, OS	Reported	52 (5–139)
Sakaguchi 2004 [62]	Japan	R	EC	120	32–74	OS	SC	60
Saito 2006 [56]	Japan	P	EEC	103	57	DFS, OS	Reported	60 (2–148)
Salvesen 1998 [58]	Norway	P	EC	96	65 (37–92)	OS	Reported	108 (60–180)
Shabani 2007 [36]	Germany	R	EC	293	64.8 (35.5–87.9)	PFS, CSS, OS	SC	89.6 (3.2–135.5)
Singh 2007 [37]	USA	P	EC	49		OS	Reported	19
Sivridis 2001 [38]	Greece	R	EC	164	–	OS	SC	55 (19–167)
Song 2012 [29]	Korea	R	EC	137	53.7 (30–82)	OS	Reported	60
Steiner 2003 [63]	Germany	R	EC	115	65 (38–81)	OS, RFS	SC	72 (36–156)
Sutton 1989 [64]	USA	R	EC	139	61 (31–89)	DFS	SC, DE	28.9 (1–128)
Voss 2011 [22]	England	P	EC	156	68.2 (37–89)	DSS, RFS	Reported	48.1 (0.1–141.5)
Zannoni 2013 [25]	Italy	P	EEC	121	59 (35–88)	DFS, OS	Reported	38 (14–91)
Zhang 2013 [53]	China	R	EC	239	54 (26–82)	DFS, OS, RFS	SC	67 (12–183)
Zou 2012 [21]	China	R	EEC	60	51.3 (30–72)	OS	Reported	45.5 (3–69.5)
C) HER2								
	Country	Study design	Disease	*N*	Age (range)	Survival analysis	Hazard ratios	Follow-up, months
Backe 1997 [54]	German	R	EC	222	68 (30–94)	OS, RFS	SC	57.6 (0.24–180)
Cianciulli 2003 [76]	Italy	R	EC	73		OS	SC	72
Coronado 2001 [72]	Spain	R	EC	114	65 (37–85)	PFS	SC	6 (14–107)
Gates 2006 [52]	USA	R	EC	99	64.2 (27–95)	OS	report	60
Gonzalez-Rodilla 2013 [20]	Spain	R	EC	126	65.9 (43–88)	OS	report	70
Jongen 2009-2 [33]	Netherlands	P	EEC	315	64.7 (32–89)	OS, RFS	Reported	59.6 (0–258)
Kohlberger 1996 [73]	Australia	R	EC	100	64 (36–85)	OS	SC	140
Konecny 2009 [68]	USA	R	EC	273	65 (38–90)	OS	SC	EEA83 (0.3–270) USPC20 (0.1–162), CCC38 (0.2–180)
Mori 2010 [67]	Japan	R	EEC	63	57.5 (32–78)	RFS, OS	SC	61.9 (7–133)
Odicino 2008 [69]	Italy	R	USPC	10	positive: 79–84; negative: 57–76	OS	DE	19.7 (1–87)
Peiro 2004 [76]	German	R	EC	10	60 (29–91)	OS	SC, reported	53
Saffari 1995 [74]	Hispanic	R	EC	75	60 (29–87)	OS	SC	144
Santin 2005-1 [70]	USA	R	USPC	27	66 (62–75)	DSS, OS	SC, reported	33 (10–48)
Santin 2005-2 [71]	USA	R	USPC	30	67.5 (63–75)	OS	SC	42 (10–51)
Togami 2012 [66]	Japan	R	UPSC	71	63.6 (47–81)	RFS, OS	Reported	49.7 (4–125)
Voss 2011 [22]	England	P	EC	156	68.2 (37–89)	DSS, RFS	Reported	48.12 (0.12–141.48)

Study design is described as prospective (P) or retrospective (R)

*EC* endometrial cancer, *EEC* endometrioid endometrial cancer, *UPSC* uterine papillary serous carcinoma, *OS* overall survival, *CSS* cancer-specific survival, *DSS* disease-specific survival, *PFS* progression-free survival, *RFS* relapse-free survival, *DFS* disease-free survival, *DE* data-extrapolated, *SC* survival curve

^−^not reported

^[ ]^Reference number

**Fig. 2:**
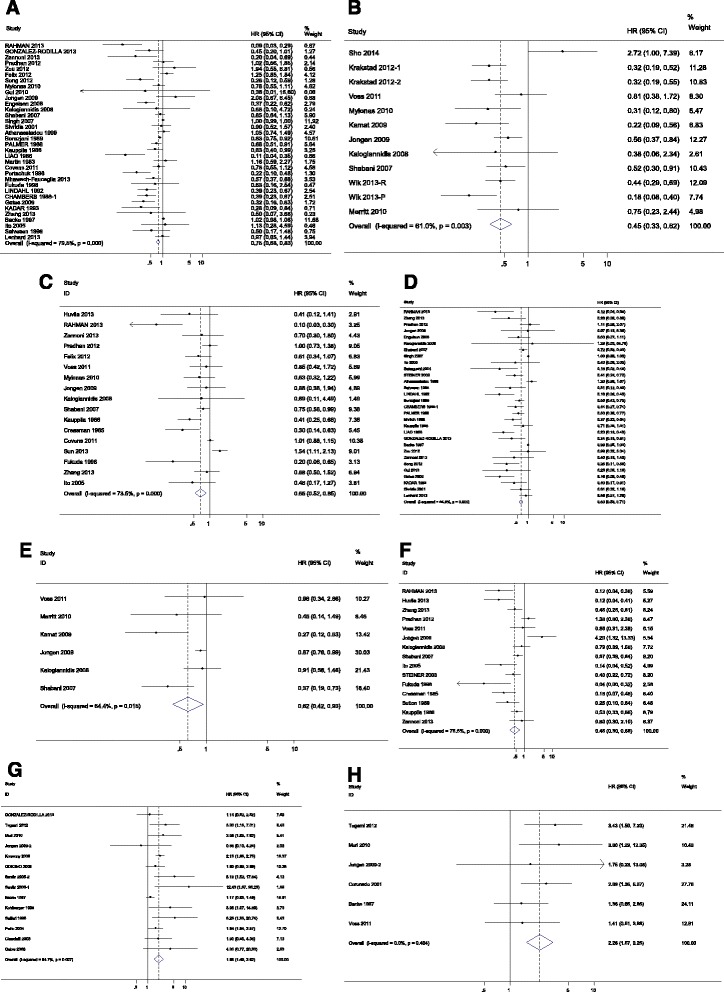
Forrest plots and meta-analysis of studies evaluating hazard ratios of high hormone receptor levels as compared to low levels in EC patients. A test of heterogeneity of combined HRs was conducted using Cochran *Q* test and Higgins I-squared statistic. Plots are arranged as follows: **a** ER OS, **b** ER CSS, **c** ER PFS, **d** PR OS, **e** PR CSS, **f** PR PFS, **g** HER2 OS, and **h** HER2 PFS

A test of heterogeneity of combined HRs was conducted using Cochran *Q* test and Higgins I-squared statistic. A *P* value of less than 0.05 was considered significant. A random-effect model (Der Simonian and Laird method) was used if heterogeneity was observed (*P* < 0.05), whereas the fixed-effect model was applied in the absence of between-study heterogeneity (*P* < 0.05). Publication bias was evaluated using the funnel plot with the Egger bias indicator test. For studies assessing EC, there mostly appeared to have heterogeneity between studies for ER, PR, and HER2 (*P* < 0.05). Hence, a random model was applied to calculate a pooled HR and its 95 % CI. Higher levels of either ER or PR could significantly indicate better survival. The pooled HRs of ER for OS, CSS, and PFS were 0.75 (95 % CI, 0.68–0.83), 0.45 (95 % CI, 0.33–0.62), and 0.66 (95 % CI, 0.52–0.85), respectively (Fig. 2a–c). The combined HRs of PR for OS, CSS, and PFS reached 0.63 (95 % CI, 0.56–0.71), 0.62 (95 % CI, 0.42–0.93), and 0.45 (95 % CI, 0.30–0.68), respectively (Fig. 2d–f). In contrast, elevated levels of HER2 could predict worse outcome with a HR of 1.98 (95 % CI, 1.49–2.62) for OS, and a HR of 2.26 (95 % CI, 1.57–3.25) for PFS (Fig. 2g, h). Such results indicated that in patients with EC, higher level of ER and PR predicted favorable survival, and increased level of HER2 was associated with poorer survival. All of the three hormone receptors had prognostic value for survival. Then, publication bias of the ERs and PRs studies were evaluated by funnel plots and Egger tests as shown in Table 2.

**Table 2:** Comparison of the predicting value of ER-α, ER-β, PR-A, and PR-B in EC patients

		OS	CSS	PFS
ER-α	HR	0.73 (0.52–1.03)	0.54 (0.30–0.98)	0.84 (0.57–1.24)
	Heterogeneity, *P* value	0.013	0.001	0.013
	Model	Fixed	Random	Fixed
	Bias, *P* value	0.379	0.968	0.975
	*N*	1568	1332	1119
	Study	7	5	6
	HR	0.90 (0.45–1.80)	–	0.84 (0.49–1.44)
ER-β	Heterogeneity, *P* value	0.847	–	0.805
	Model	Fixed	–	Fixed
	Bias, *P* value	0.771	–	0.287
	*N*	925	–	925
	Study	4	–	4
	HR	1.00 (0.99–1.00)	–	0.78 (0.18–3.44)
PR-A	Heterogeneity, *P* value	0.066	–	0.001
	Model	Fixed	–	Fixed
	Bias, *P* value	0.026	–	0.652
	*N*	1038	–	696
	Study	5	–	3
	HR	0.67 (0.49–0.90)	–	0.60 (0.43–0.82)
PR-B	Heterogeneity, *P* value	0.841	–	0.656
	Model	Random	–	Random
	Bias, *P* value	0.748	–	0.32
	*N*	696	–	696
	Study	3	–	3

A test of heterogeneity of combined HRs was conducted using Cochran *Q* test and Higgins I-squared statistic. A random-effect model (Der Simonian and Laird method) was used if heterogeneity was observed (*P* < 0.05), whereas the fixed-effect model was applied in the absence of between-study heterogeneity (*P* < 0.05). Publication bias was evaluated using the funnel plot with the Egger bias indicator test

*EC* endometrial cancer, *ER-α* estrogen receptor-alpha, *ER-β* estrogen receptor-beta, *PR-A* progesterone receptor-A, *PR-B* progesterone receptor-B, *HR* hazards ratio, *OS* overall survival, *CSS* cancer-specific survival, *DSS* disease-specific survival, *PFS* progression-free survival, *DFS* disease-free survival, *RFS* relapse-free survival

Previous studies reported that two distinct receptors(ER-α and ER-β) may exert opposite effects on cellular processes that include proliferation, apoptosis, and migration, and their different effects may depend on tumor type and disease stage [77]. Considering that the different subtypes of ER and PR may have different effects on cancer survival, we identified that the studies focusing on ER-α, ER-β, PR-A, and PR-B performed a meta-analysis. The pooled HRs of ER-α for OS, CSS, and PFS were 0.73 (95 % CI, 0.52–1.03), 0.54 (95 % CI, 0.30–0.98), 0.84 (95 % CI, 0.57–1.24), respectively. The combined HRs of ER-β for OS and PFS were 0.90 (95 % CI, 0.45–1.80) and 0.84 (95 % CI, 0.49–1.44). The pooled HRs of PR-A for OS and PFS were 1.00 (95 % CI, 0.99–1.00) and 0.78 (95 % CI, 0.18–3.44). The combined HRs of PR-B for OS and PFS were 0.67 (95 % CI, 0.49–0.90) and 0.60 (95 % CI, 0.43–0.82). The results are summarized in Table 2.

**Fig. 3:**
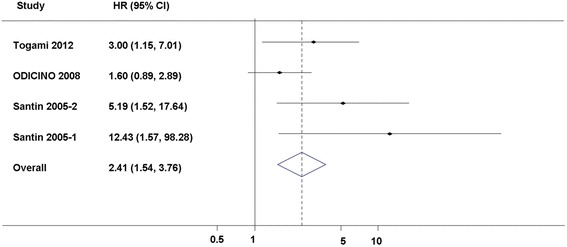
Forrest plots and meta-analysis of studies evaluating hazard ratios of HER2 levels as compared to low levels about OS in UPSC patients. A test of heterogeneity of combined HRs was conducted using Cochran *Q* test and Higgins I-squared statistic

The pathogenetic role and prognostic value of HER2 in EC, especially in uterine papillary serous carcinomas [78], one of the most malignant histological types of EC, have recently become the focus of several studies, providing the molecular basis for targeted immunotherapy against the highly aggressive tumors [66, 69, 79–84]. Then we tried to identify the studies focusing on uterine papillary serous carcinoma (UPSC) and performed a meta-analysis. Although there were only four studies (*n* = 138) that could be included in this subgroup meta-analysis, the pooled HR was 2.41 with 95 % CI from 1.54 to 3.76 (*P* < 0.05) for OS [66, 69–71] (Fig. 3). The HR was significant, and it was potentially strong as a HR of an empirical cutoff for strong predictor [84].

## Conclusions

This meta-analysis indicated that hormone receptors may have value in predicting survival in patients with endometrial cancer. The higher levels of ER and PR were significantly associated with favorable survival, whereas the increased level of HER2 predicted poorer survival. All of the three hormone receptors had prognostic value for survival. ER and PR expression are used to identify endometrial cancer (EC) patients that could benefit of hormone therapy, and there are many evidences suggesting that they can be good biomarkers predicting hormone therapy response, but further validation will be required before they are incorporated in routine management of EC patients.

However, this meta-analysis has several limitations and the conclusions should be tempered. First, marked heterogeneity of subjects existed in distinct groups. The heterogeneity of the population was probably due to the difference in the baseline characteristics of patients (age, tumor stage, race, methodology for assessing HRs expression, or country), the cutoff value of markers, the undergoing treatment, the duration of follow-up, and others. To minimize the residual confounding effect caused by the heterogeneity within these studies, a random-effect model was applied. Furthermore, publication bias was detected in all the meta-analyses, and this cannot be adequately overcome by currently available statistical techniques. In addition, although the result of UPSC subgroup about HER2 was promising, the conclusion should be tempered for the relatively small sample size.

Steroid hormones, including ovarian steroid hormones progesterone and estrogen, play vital roles in the development of benign endometrium and endometrial cancer via their receptors [85]. Estrogens act as a promoter of growth and proliferation of the endometrium via estrogen receptors, while progesterone acts as an estrogen antagonist in endometrial maturation and inhibition of proliferation [86]. The endometrium is very sensitive to sex hormones, and thus a shift in the balance of estrogens and progesterone can cause the development of endometrial cancer [1]. The glandular epithelium from which the cancer arises is hormone responsive, expressing both PRs (PR-A and PR-B) and ERs (ER-α and ER-β) [87].

EC often develops from endometrial hyperplasia, which is attributed to prolonged exposure to estrogen in the absence of (unopposed) sufficient progesterone [88], and is often well differentiated and non-invasive or superficially myoinvasive, rarely producing metastases and expressing ER [89]. Whereas early-stage, well differentiated EC usually retain expression of both receptors, advanced stage, poorly differentiated tumors often lack one or both of these receptors, which has been correlated in many studies with a poor prognosis [19, 47]. In our meta-analysis, both ER and PR tend to be linked with favorable outcome of endometrial cancer and could be applied as a significant predictor. Our results were consistent with most of the previous basic studies that suggested the protective role of PR in endometrial cancer.

Estrogens stimulate cell proliferation through the classical estrogen receptors ER-α and ER-β. ER-α and ER-β have a distinct pattern of expression in the tissues [90], which varies during cellular proliferation and differentiation [91]. Usually ER-α was the dominant isoform in specimens of normal and diseased endometrium [92, 93]. Some recent studies revealed that ER-α was associated with aberrant proliferation, inflammation, and the development of malignancy, whereas ER-β seemed to oppose ER-α actions on cell proliferation by modulating the expression of many ER-α-regulated genes and exhibits anti-migratory and anti-invasive properties in cancer cells [77]. In large cohorts of EC patients, ER-α was related to early stage, lower-grade tumors [17, 33], whereas ER-β was related to late stage EC [94]. Our study also conducted a meta-analysis about different ERs, but elevated ER-α and ER-β levels alone had no significant value in predicting favorable survival than non-distinguished ER. Therefore, we suggested more studies on ER-α and ER-β in the future to further clarify the distinct role of ERs and PRs in the development of endometrial carcinoma and to also help identify diagnostic or therapeutic markers.

The single-copy PR gene uses separate promoters and translational start sites to produce two isoforms, PR-A and PR-B [95], which are in fact two functionally distinct transcription factors [96] and mediate their own response genes [95, 97–99]. Studies in mice with selective ablation of PR isoforms revealed that PR-A is necessary for ovulation and modulates the anti-proliferative effects of progesterone in the uterus and that PR-B is required for normal mammary gland development and function [100, 101]. To date, there is no evidence of such selective roles of PR-A and PR-B in human tissues. Clinical data in relation to the prevalence of steroid receptor isoforms PR-A and PR-B are scarce, and the specific mechanism is unclear. In our current meta-analysis, elevated ER-α, ER-β, and PR-A levels did not reach significant level majorly due to the limited study number and sample size. The polled HR of PR-B was associated with better outcome, but there were only three studies that could be included in this subgroup meta-analysis. Further analysis in large scale study may contribute to the understanding of ER and PR isoforms expression in EC.

In addition, HER2 plays a crucial role in the growth of both normal tissue and malignant tumors [11]. HER2 amplification and overexpression have been shown to play a key role in the pathogenesis of various different cancer types, including breast, ovarian, gastric, and esophageal carcinomas [102].

HER2 overexpression was also found to be associated with endocrine therapy resistance, and HER2-positive cancer might have a worse clinical outcome [103]. Our study has demonstrated the predictive role of elevated HER2 level for poorer survival. Such data may indicate the harmful role of HER2 in endometrial cancer.

In summary, both elevated level of ER and PR predicted favorable survival, and elevated level of HER2 was associated with worse survival in endometrial cancer. The association between hormone receptor status and survival raises the possibility of different subsets of 3patients with endometrial cancer with different biologic behavior and different response to treatment but similar histology or similar clinical performance. Conventional histological examination alone may not be enough to guide therapy and to refine the outcome prediction. We suggest examining ER, PR, and HER2 levels to evaluate endometrial cancer prognosis.

## Abbreviations

EC: Endometrial cancer
ER: Estrogen receptor
HER2: Human epidermal growth factor receptor 2
OS: Overall survival
CSS: Cancer-specific survival
DSS: Disease-specific survival
PFS: Progression-free survival
DFS: Disease-free survival
RFS: Relapse-free survival
PR: Progesterone receptor
UPSC: Uterine papillary serous carcinoma
